# Prognostic risk score development to predict birth asphyxia using maternal and fetal characteristics in South Gondar zone hospitals, north West Ethiopia

**DOI:** 10.1186/s12887-022-03582-x

**Published:** 2022-09-10

**Authors:** Desalegn Tesfa, Sofonyas Abebaw Tiruneh, Melkalem Mamuye Azanaw, Alemayehu Digssie Gebremariam, Melaku Tadege Engidaw, Mulu Tiruneh, Tsion Dessalegn, Melkamu Aderajew Zemene, Ermias Sisay

**Affiliations:** 1 Department of Public Health, College of Health Sciences, Debre Tabor University, Debre Tabor, Ethiopia; 2 Department of Pediatrics & Child Health, College of Health Sciences, Debre Tabor University, Debre Tabor, Ethiopia

**Keywords:** Prediction, Prognostic risk score, Birth asphyxia, Decision curve

## Abstract

**Background:**

Birth asphyxia leads to profound systemic and neurological sequela to decrease blood flow or oxygen to the fetus followed by lethal progressive or irreversible life-long pathologies. In low resource setting countries, birth asphyxia remains a critical condition. This study aimed to develop and validate prognostic risk scores to forecast birth asphyxia using maternal and neonatal characteristics in south Gondar zone hospitals.

**Methods:**

Prospective cohorts of 404 pregnant women were included in the model in south Gondar Zone Hospitals, Northwest Ethiopia. To recognize potential prognostic determinants for birth asphyxia, multivariable logistic regression was applied. The model discrimination probability was checked using the receiver operating characteristic curve (AUROC) and the model calibration plot was assessed using the ‘givitiR’ R-package. To check the clinical importance of the model, a cost-benefit analysis was done through a decision curve and the model was internally validated using bootstrapping. Lastly, a risk score prediction measurement was established for simple application.

**Results:**

Of 404, 108 (26.73%) (95% CI: 22.6–31.3) newborns were exposed to birth asphyxia during the follow-up time. Premature rupture of membrane, meconium aspiration syndrome, malpresentation, prolonged labor, Preterm, and tight nuchal was the significant prognostic predictors of birth asphyxia. The AUROC curve for birth asphyxia was 88.6% (95% CI: 84.6-92.2%), which indicated that the tool identified the newborns at risk for birth asphyxia very well. The AUROC of the simplified risk score algorithm, was 87.9 (95% CI, 84.0– 91.7%) and the risk score value of 2 was selected as the optimal cut-off value, with a sensitivity of 78.87%, a specificity of 83.26%, a positive predictive value of 63.23%, and a negative predictive value of 91.52%.

**Conclusions:**

We established birth asphyxia prediction tools by applying non-sophisticated maternal and neonatal characteristics for resource scares countries. The driven score has very good discriminative ability and prediction performance. This risk score tool would allow reducing neonatal morbidity and mortality related to birth asphyxia. Consequently, it will improve the overall neonatal health / under-five child health in low-income countries.

## Introduction

According to the world health organization (WHO) birth asphyxia, neonatal asphyxia, or perinatal asphyxia is defined as the “inability of the newborn to initiate and sustain spontaneous breathing at birth” [1, 2]. Birth asphyxia is determined by the parameter of Apgar score assessed in the first and fifth minute of delivery, with a score ranging from zero to ten [3]. The one and five-minute Apgar scores measure how well the newborn tolerated the breathing processes and how well the newborn adapted to the environment [4]. A range of 0-3, 4-6, and 7-10 indicates low, moderate, and normal Apgar scores [3, 4].

Globally, birth asphyxia/perinatal asphyxia is a foremost long-term consequence in adulthood characterized by an increased risk of cardiovascular diseases [1], causing 24% of all neonatal deaths [5] and the third leading cause of under-five child deaths (11%) succeeding premature birth (17%) and pneumonia (15%) [6]. However, in Ethiopia, in 2015, birth asphyxia was the primary cause of neonatal mortality (31.6%) followed by prematurity (21.8%) and infection (18.5%) [7]. Particularly, the Amhara regional state of Ethiopia accounts for the uppermost neonatal death rate (47 per 1000 live births) as compared to other regions of Ethiopia, Tigray, Afar, Oromia, Somali, Benishangul-Gumuz, South Nations-Nationality, Gambela, Harari, Addis Abeba, and Drie Dwa with a mortality rate of 34, 38, 37, 41, 35, 35, 36, 34, 18, and 36 per 1000 live birth respectively [8].

Oxygen deficit at delivery is the leading cause of brain damage which can lead to severe insults often causing neurodegenerative disease, mental retardation, and epilepsy [9, 10]. The mild insults lead to attention deficit, hyperactivity, development of schizophrenia, and lifelong functional psychotic syndrome [4, 5]. Birth asphyxia is accompanied by a multifaceted variety of risk factors whether they are antepartum, intrapartum, or postpartum [11]. However, the quality of intrapartum care during labor and delivery has been documented as the solitary most significant factor in indisposition and death from asphyxia neonatorum [12].

Risk factors before delivery include severe maternal hypotension or hypertensive disorders while pregnancy [13], Antepartum hemorrhage [14], previous stillbirth [15], maternal anemia [13, 16] young and progressive maternal period [13], and low educational attainment [15]. Intrapartum risk Factors: mal-presentation [13], prolonged second stage labor [14, 16] and meconium-stained amniotic fluid [17–22]. Fetal risk factors include low birth weight and, numerous gestation, close-fitting nuchal cord [14, 23], premature delivery [13], and fetal suffering from oxygen deprivation [15].

Currently, the 2030 sustainable development goals agenda 2030, combines multisystem strategies at the international and national level and has three essential focuses to realize healthy lives and encourage welfare for all ages. Of those goals, one leading aim is to decrease the death rate of neonates to less than 12 per 1000 live births [6, 24]. Nevertheless, even though the application of numerous implementable strategies, approaches, and interventions are designed for the maternal and neonatal side to reduce child mortality, currently under-five child death, infant, and neonatal mortality because of birth asphyxia remain highest in developing countries [5, 6, 25].

For better clinical care and public health intervention, understanding the risk probability of birth asphyxia is too essential. In the clinical setting, prediction can be used for the adjustment of lifestyle or therapeutic decisions based on the risk of developing a specific consequence [26]. A convenient and easily applicable clinical prognosis is a very vital tool to predict birth asphyxia. Therefore, the developed risk score could be important for clinicians (especially, gynecologists, midwives, pediatric residents, neonatal nurses, and neonatologists) to integrate several patient characteristics and symptoms (determinants, test results, etc) to make a future prediction [16].

Therefore, the prediction model is inherently multivariable. Prediction models (also commonly called “prognostic models”, “risk scores,” or “prediction rules”) are tools that combine multiple predictors by assigning relative weights to each predictor to obtain a risk or probability condition to happen [14]. Multivariable prediction models in high-income countries may use advanced laboratory biomarkers. However, in low-resource countries using advanced and sophisticated laboratory biomarkers is impossible. Developing and validating prognostic risk scores using the most important predictors is critical. Therefore this study aimed to develop and validate important clinical and prognostic risk scores to predict birth asphyxia in South Gondar zone Hospitals, Northwest Ethiopia.

## Methods and materials

### Study setting

This study was carried out among pregnant women those followed antenatal care in four Hospitals in the South Gondar zone (Debre Tabor, Nefas- Mewicha, Mekane Eyesus, and Addis Zemene) [27]. Based on 2019, South Gondar Zone report, this zone has a total population of 2, 609,824. Of these, around 527,967 reproductive age group females and 87,955 pregnant women were found in the zone.

The zone has 8 Hospitals, 96 health centers, 394 health posts, and 118 private health institutions. All of the hospitals give similar services except Debre Tabor general hospital which serves as a referral center for -private and governmental institutions [27].

#### Study design

An institution-based prospective follow-up study was conducted among pregnant mothers from June 1st, 2020 to June 30th, 2021.

#### Inclusion

in this study, all selected pregnant women who followed antenatal care in four hospitals (Nefas- Mewicha, Debre Tabor, Mekane Eyesus, and Addis Zemene Hospitals) from June 1st, 2020 to June 30th, 2021 were included.

#### Exclusion

women couldn’t answer the intended questions because of illness and mental problems were excluded.

### Study participants and data collection procedure

Since there are no previous prediction studies to estimate the sample size, considering the rule of thumb of 10 events per prognostic determinants, [28], all pregnant women who were enrolled in the cohort (from June 1st, 2020 to June 30th, 2021) and fulfilled the eligibility criteria were included in the analysis. In this period, 410 pregnant women were followed, but unfortunately, since 6 of the women didn’t give live birth, 404 women (give live birth) were included. This means that only 404 newborns were considered To increase the potential for developing a robust prediction model, the sample size should be at least large enough to minimize model overfitting and to target sufficiently precise model predictions. For this study, a total of fourteen (current maternal age, maternal education, gravidity, the premature rupture of membrane, meconium aspiration syndrome, malpresentation, prolonged labor, preeclampsia, tight nuchal cord, preterm, have chronic hypertension, hemoglobin level, sex of the child, and birth weight) maternal and neonatal prognostic determinants were considered.

Data were collected prospectively using pre-tested structured questionnaires. Questionnaires were developed after reviewing relevant literature to include all the possible variables that address the objective of this study. Firstly, the questionnaire was developed in English and translated into the local language (Amharic), and finally, retranslated into English by a language expert. For each Hospital four clinical midwifery health professionals participated as data collectors.

### Operational definition

#### Birth asphyxia

Birth asphyxia was defined as an Apgar score of < 7 at the 5th minute with five signs (activity, Pulse, grimace, appearance, and respiration) each of them has 0 to 2 scores [4, 29, 30].

#### Prolonged second stage

Defined according to r American College of Obstetricians and Gynecologists guidelines as: for nulliparous women > 3 hours with epidural or > 2 hours without; multiparous women > 2 hours with epidural or > 1 hour without. The length of the second stage of labor was determined by subtracting the date and time of delivery from the date and time of 10 cm cervical dilation [29, 30].

#### Nuchal cord

Nuchal cord was defined as a loop of the umbilical cord that becomes wrapped around the fetal neck 360 degrees. During delivery, each birth was documented as having a tight nuchal cord, or no tight nuchal cord. ‘tight nuchal cord’ was defined as the inability to manually reduce the loop over the fetal head [29].

#### Premature rupture of membrane

was identified when fetal Chorio-amnionic membranes rupture at any time before the beginning of factual labor [4, 30].

#### Meconium aspiration syndrome (MAS)

this happens after the fetus inhales profuse, particulate meconium. This is foremost secondary to fetal hypoxia which causes amplified peristalsis, relaxation of anal sphincters with the release of meconium into the amniotic fluid; and reflex grasping which leads to aspiration of the meconium into the respiratory system [4, 30].

### Variables

#### Dependent variable

Birth asphyxia (yes/no).

#### Prognostic determinants

current maternal age maternal education, gravidity, the premature rupture of membrane, meconium aspiration syndrome, malpresentation, prolonged labor, preeclampsia, tight nuchal cord, preterm, have chronic hypertension, hemoglobin level, sex of the child, and birth weight prognostic maternal and neonatal determinants were considered.

### Data processing and analysis

Before data was entered to EPI INFO windows –version 7 statistical software, all the entire interviewed questionnaires were checked manually for completeness and consistency. After that, the data were entered, coded, and cleaned. Finally, the entered data were transferred to R-software for further analysis. Descriptive statistics such as frequency and percentage were performed. Bivariate and multivariable logistic regression was performed to identify potential prognostic determinants. Simple logistic regression was conducted on the derivation data set to investigate the relationship between each predictor and birth asphyxia. We apply the stepwise backward elimination technique with a *p*-value < 0.10 for the likelihood ratio test to fit the reduced model of easily obtainable determinants. The results were reported as an adjusted odds ratio with 95% CI at a two-sided *P*-value of less than 0.05.

### Assessment of the model performance and validation

Model calibration was estimated by plotting the predicted probability against the observed birth asphyxia. Consequently, the model calibration was checked using the ‘givitiR’ R-package. The area under the receiver operating characteristics curve (AUROC) was conducted using the ‘pROC’ R-package to check the model discrimination probability. The AUROC value of 0.5, 0.7, and 1 indicates no discriminative probability, good discriminative probability, and perfect discrimination probability respectively. The internal validation was checked using bootstrapping technique with 2000 iterations of re-samplings. After bootstrapping model’s predictive performance is considered to apply to future similar populations. Through the decision curve, the cost-benefit analysis of the prediction tool was checked.

### Risk score development

To develop a straightforward, implementable, and interpretable risk score for birth asphyxia, each estimate (*β* coefficient) is divided by the lowest *β* value of the individual variable and rounded to the nearest integer.

The predicted probability of birth asphyxia was presented according to two categories of the risk score. To realize the risk score cut point, we apply the Youden index value (sensitivity + specificity − 1) of each category on the risk. Later, the score was transformed into a prediction test classified as high risk or low risk of birth asphyxia. The risk score instrument specificity, sensitivity, and positive and negative predictive value were determined at different values of the risk score cut-points. Finally, using each prognostic determinant risk score value, the probability of birth asphyxia was determined.

## Results

Overall, a total of 404 pregnant women before delivery and 404 neonates after delivery were followed prospectively for this study. Around two-thirds, 258(63.86%) of the women were found in the age category of 20-29 years, and around one-third of 125 (30.94%) of their educational status were able to read and write. One hundred twenty-one (29.95%) pregnant women were exposed to prolonged labor during labor. One hundred fifteen (28.47%) newborn babies were exposed to meconium aspiration syndrome and fifty-five (13.6%) newborn babies were delivered with low birth weight (Table 1).

**Table 1:** Characteristics of the study participants

Prognostic determinants	Frequency	Percent
**Current maternal age**		
≤ 19 years	32	92
20-29 years	258	63.86
30-39 years	105	25.99
≥ 40 years	9	2.23
**Maternal educational status**		
Unable to read and write	111	27.48
Able to read and write	125	30.94
Primary	47	11.63
Secondary	68	16.83
College and above	53	13.12
**Gravidity**		
≤ 2	176	43.56
3-4	170	42.08
≥ 5	58	14.36
**Premature rupture of membrane**		
No	331	81.93
Ye	73	18.07
**Meconium aspiration syndrome**		
No	289	71.53
Yes	115	28.47
**Mal presentation**		
No	327	80.94
Yes	77	19.06
**Prolonged second stage labor**		
No	283	70.05
Yes	121	29.95
**Preeclampsia**		
No	359	88.86
Yes	45	11.14
**Tight nuchal cord**		
No	335	82.92
Yes	69	17.08
**Preterm**		
No	332	82.18
Yes	72	17.82
**Have chronic hypertension**		
No	362	89.60
Yes	42	10.40
**Hemoglobin level**		
> 11 g/d	333	82.43
≤ 11 g/dl	71	17.57
**Sex of the child**		
Male	172	42.57
Female	232	57.43
**Birth weight**		
> 2500 g	349	86.39
≤ 2500 g	55	13.61

### Birth asphyxia predictive model

Maternal and neonatal clinical characteristics were included for the prediction of birth asphyxia. In the bivariable model, the premature rupture of membrane, meconium aspiration syndrome, malpresentation, prolonged labor, Birth weight, Preterm, tight-nuchal cord, hemoglobin level, and time of delivery are significant predictors of birth asphyxia. However, after the reduced multivariable model, the premature rupture of membrane, meconium aspiration syndrome, malpresentation, prolonged labor, Preterm, and tight nuchal, were the prognostic predictor of birth asphyxia (Table 2). The probability of birth asphyxia prediction based on the linear prediction using the regression formula is:

**Table 2:** Prognostic determinants of birth asphyxia

Determinant variables	Birth asphyxia		Bivariable (COR, 95% CI)	Multivariable (AOR,95%CI)	Risk score
	No	Yes			
**Premature rupture of membrane**					
No	276	55		1	2
Yes	20	53	13.30(7.48-24.47)	5.43(2.59-11.60)	
**Meconium aspiration**					
No	239	50		1	1
Yes	57	58	4.86(3.03-7.87)	3.12(1.66-5.89)	
**Mal presentation**					
No	273	54	1	1	1
Yes	23	54	11.87(6.81-21.30)	4.60(2.26-9.43)	
**Prolonged second stage labor**					
No	236	47	1	1	1
Yes	60	61	5.11(3.19-8.25)	3.65-1.98-6.80)	
**Birth weight**					
> 2500 g	266	83	1		1
≤ 2500 g	30	25	2.67(1.48-4.80)		
**Preterm**					
No	264	68	1	1	1
Yes	32	40	4.85(2.85-8.34)	3.82(1.86-7.90)	
**Tight nuchal cord**					
No	266	69	1	1	1
Yes	30	39	5.01(2.92-8.70)	3.18(1.57-6.45)	
**Hemoglobin level**					
> 11 g/dl	262	71	1		
≤ 11 g/dl	34	37	4.01(2.36-6.88)		
**Time of delivery**					
Day	197	64	1		
Night	99	44	1.37(1.01-2.20)

$$\mathrm{Linear}\;\mathrm{prediction}\;\mathrm{of}\;\mathrm{the}\;\mathrm{model}\;(\mathrm{lP})\:=\:-\:3.17\;+\;1.69\ast\mathrm{premature}\;\mathrm{rapture}\;\mathrm{of}\;\mathrm{membrane}\;+\;1.14\ast\mathrm{meconium}\;\mathrm{aspiration}\;+\;1.53\ast\mathrm{mal}-\mathrm{presentation}\;+\:1.29\ast\mathrm{prolonged}\;\mathrm{labor}\;+\:1.34\ast\mathrm{preterm}\;+\:1.16\ast\mathrm{tight}\;\mathrm{nuchal}\;\mathrm{cord}.\;\mathrm{Therefore}\;\mathrm{the}\;\mathrm{probability}\;\mathrm{of}\;\mathrm{birth}\;\mathrm{asphyxia}\;\mathrm{will}\;\mathrm{be}\;\mathrm{the}\;\mathrm{regression}\;\mathrm{formula}\;\mathrm{equal}\;\mathrm{to}\;\mathrm P/\;\mathrm{birth}\;\mathrm{asphyxia}\;=\;\exp^{(lp)}/(1\:+\:exp^{(lp)}).$$

### Discrimination

**Fig. 1:**
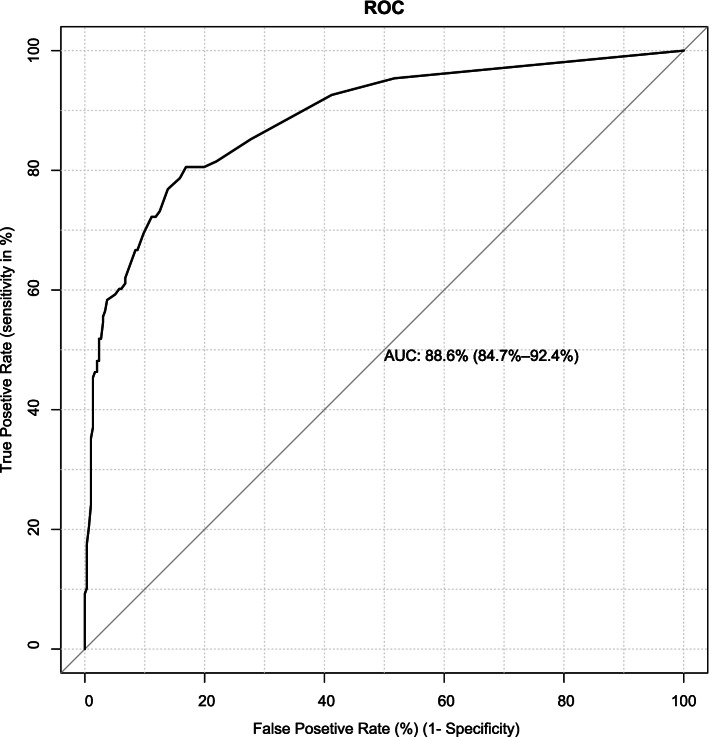
Area under the receiver operative characteristic curve of the reduced model

The discriminatory power of the model was assessed by the AUC of the ROC curve plotting sensitivity against the 1-specificity of the model (Fig. 1).

**Fig. 2:**
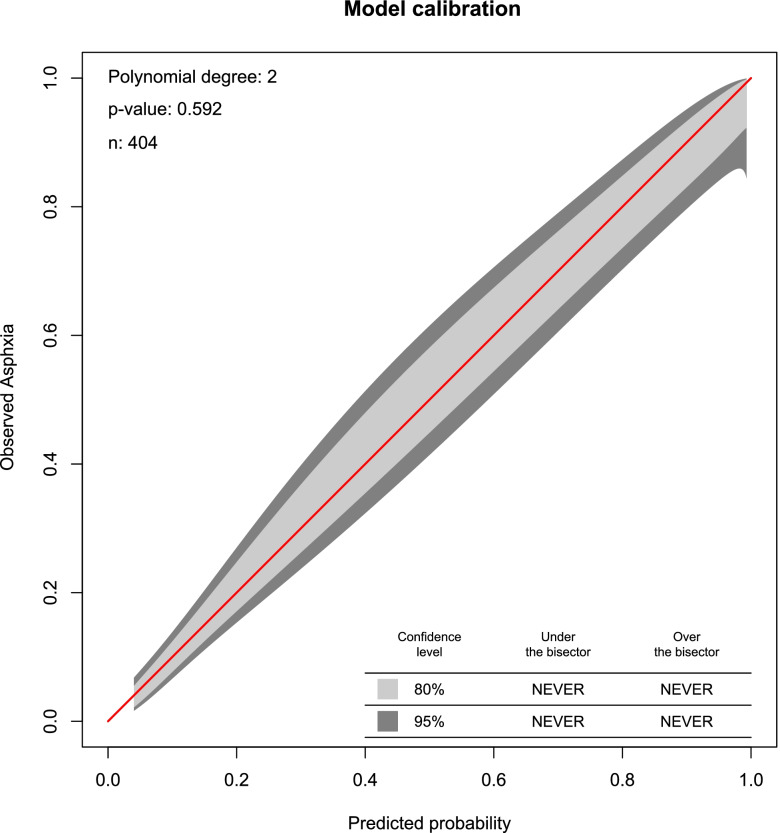
Model calibration plot for predicted birth asphyxia

**Fig. 3:**
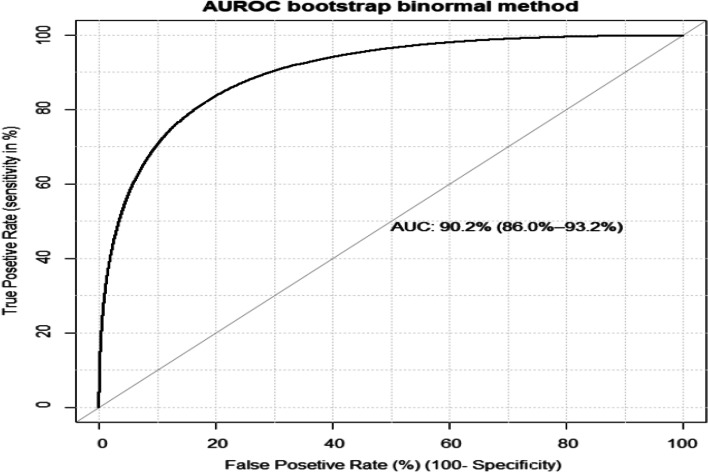
Area under the receiver operating characteristics after internal validation

The AUROC was 88.6% (84.6-92.2% for the reduced model which is a good discriminative probability The model calibration was checked by comparing agreement between the predicted probability of birth asphyxia and observed birth asphyxia using a calibration plot (*p*-value =0.592 (Fig. 2). After internal validation by 2000 bootstrap replicates, the 95% confidence interval of the AUROC curve was 90.2%% (95% CI: 86. 0 - 93.2%) (Fig. 3).

### Decision curve analysis of the model

**Fig. 4:**
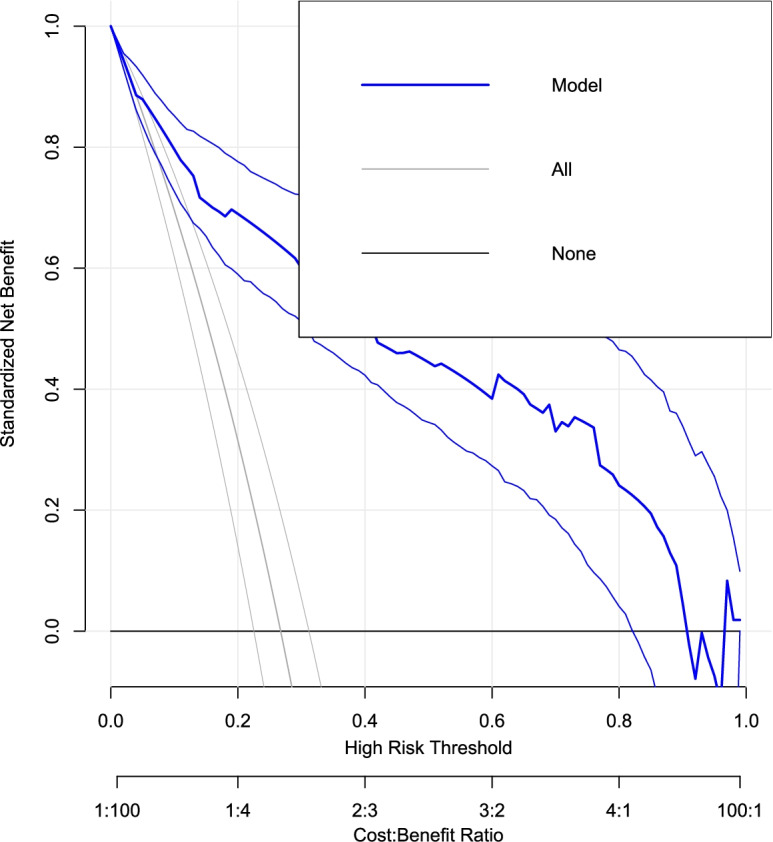
Decision curve analysis for cost-benefit analysis

Across the entire range of thresholds, the model has a very good highest net benefit ratio (Fig. 4). This indicated that it is of greater public health importance. This model predicts the highest likelihood of birth asphyxia. As a result, regardless of the risk threshold, early recognition should be the primary activity because the model has a higher net benefit ratio than not using (referring) at all or referring to all..

### Clinical prediction and decision rules for birth asphyxia

**Fig. 5:**
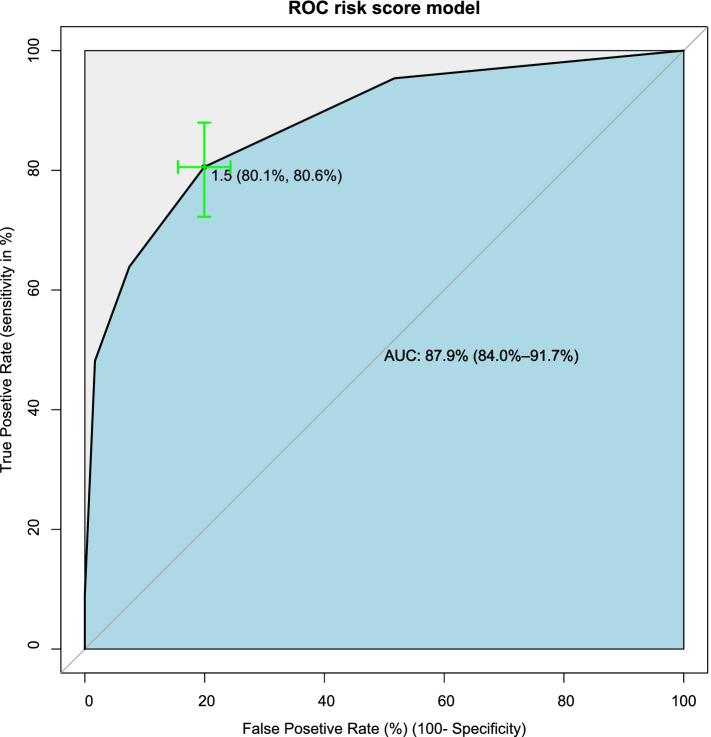
Area under the receiver operating characteristics curve and Youden index cut-point for the risk score tool

For the comfort of prognostic application, a score chart rule was applied for the decision to high or low risk. By this, the prediction of the risk score tool had 7 scores. The AUROC of simplified risk score was 87.9% (95% CI; 84.0-91.7) (Fig. 5), which indicated that the tool identified the neonate at risk for birth asphyxia very well. The cut-off value was chosen to maximize the sensitivity and specificity, aiming to minimize the number of false positives and false negatives. For clinical decision-making risk, the score is categorized as low risk and high risk of birth asphyxia, and this risk score cut point was declared as using youden’s index value which had the maximum sensitivity and specificity of the risk score. At the cut point of 1.5 the sensitivity and the specificity of the risk score ROC curve were maximized (Fig. 5).

Subsequently, the individual prediction of birth asphyxia was a high risk if the newborns have a risk score value of more than and equal to two (after circumnavigating to the nearest integer). Based on the risk category, 258 newborn babies had a risk score of less than 2; of them, 21 (8.1%) were exposed to birth asphyxia. However, 146 had a risk score of ≥2, the 87 (59.6%) experienced birth asphyxia. The sensitivity and the specificities were 78.87 and 83.26% respectively with a positive predictive value of 63.23% and negative predictors of 91.52% (Table 3).

**Table 3:** prognostic risk classification of birth asphyxia using a simplified prediction risk score

Risk category	Score range	prediction of birth asphyxia					
		No birth asphyxia	birth asphyxia	SN	SP	PPV	NPV
**Low risk**	< 2	258 (63.9)	21(8.1)	78.87	83.26	63.23	91.52
**High risk**	≥2	146 (36.1)	87(59.6)				
**Total**	7	404(100%)	108 (26.73%)				

Probability of birth asphyxia = (2*Premature rapture of membrane) + (1* Meconium aspiration) + (1* Mal presentation) + (1* Prolonged labor) + (1*preterm) + (1* Tight nuchal cord).

## Discussion

The present study reviled that the incidence of birth asphyxia was 26.7%. In this study, the most prominent aim was to develop and validate a simplified clinical prognostic risk score system for birth asphyxia using 7 maternal and neonatal characteristics during pregnancy without the need for any sophisticated laboratory or imaging test. The combination of six maternal and neonatal characteristics produces a true prediction accuracy of 0.86, which is a sweeping tool to predict birth asphyxia according to diagnostic accuracy classification [31].

To achieve the highest aspiration model, premature rupture of membrane, meconium aspiration syndrome, malpresentation, prolonged labor, tight nuchal cord, and preterm was the prognostic determinant of birth asphyxia. A clinical assessment and laboratory examinations are required to evaluate and manage the asphyxiated newborn before the problem advanced to irreversible organ damage and death. The American College of Obstetricians and Gynecologists and the American Academy of Pediatrics allocate a neonate to be asphyxiated if the succeeding circumstances are satisfied: Umbilical cord arterial pH < 7; Apgar score of 0-3 for longer than 5 min; neurological manifestations (e.g., seizures, coma, or hypotonia); and multisystem organ dysfunction, e.g., cardiovascular, gastrointestinal, hematological, pulmonary or renal system. At the same time, the overhead standards could be valuable and valid in developed countries, however, they cannot be practically made use of in resource-limited countries [32].

Therefore, this study offers an enduring solution (appropriate risk score measurement) using the Youden index cut-off point, which has a supreme true positive rate (sensitivity) and true negative rate (specificity).

This prediction model has an outstanding negative predictive value and true negative rate (specificity), signifying that an advantageous preliminary screening tool to recognize the jeopardy of the neonate for birth asphyxia. This is the primary study that develops and validates an early warning risk score tool for the prediction of birth asphyxia in low-income countries. For this current prediction study, the premature rupture of the membrane has been a high predictive value for birth asphyxia. Simultaneously this finding was supported by several studies which were conducted in Colombia [33], Al-Diwaniya [34], Cameroon [35], and Uganda [18].

The agreement could be accepted the fact that whiles the fetal membrane ruptured impulsively, extemporaneous amniotic liquid occurs lengthways with umbilical cord prolapse and compression. The longer the distance of the rapture of membranes, the more likely the uterus increases the incidence of fetal and maternal morbidity suffering, and mortality [36]. Meconium aspiration syndrome was the additional prognostic determinant of birth asphyxia, which was analogous to the various studies conducted in the Swedish urban population [17], Uganda [18], Ethiopia (in different study areas and study populations) [19–22].

The conceivable explanation, the occurrence of meconium in the amniotic fluid may cause aspiration of meconium-stained amniotic fluid to happen, and this can block minor airways, deactivate surfactants, chemical inflammation, and apoptosis of the pulmonary tissues, and may also constrain surfactant production, causing in a pulmonary air leak and birth asphyxia [37]. Like other variables, a mother encountering prolonged second-stage labor was one of the prognostic determinants of birth asphyxia in this study. Hence, it was supported by studies accompanied in Ethiopia [20] and Colombia [33]. This might be the reason that babies born after extended labor have a larger danger of birth asphyxia and birth trauma leads to umbilical cord difficulties or the stress of too many contractions. Prematurity was another prognostic determinant of birth asphyxia, which was in line with a study found in Ethiopia [14, 22] and Pakistan [23].

Babies being born earlier than 37 completed weeks of gestation may be exposed to birth asphyxia due to an unsatisfactory amount of surfactant in the lungs [38]. Surfactant generates an uninterruptedly reforming surface layer over the alveoli which diminishes surface tension, prevents atelectasis, and maintains alveolar stability. A deficiency in pulmonary surfactant production causes respiratory distress syndrome [38, 39]. The furthermore tight nuchal cord was a prognostic determinant of birth asphyxia. Previous studies evidenced that the tight nuchal cord was a strong substantial risk factor for birth asphyxia [20, 21, 29]. This might be related to tight nuchal cords that can interject usual blood, nutrient, and oxygen exchange by compressing the umbilical cord or limiting arteries and veins in the fetal neck, which can lead to birth asphyxia [40].

Lastly, malpresentation was one of the prognostic determinants of birth asphyxia. Consequently, this study was supported by [20, 41]. The possible justification could be because malpresentation is often associated with premature rupture of membrane, a prognostic determinant in this study. Succeeding premature rupture of the membrane, newborn life intimidating occasions like umbilical cord accidents (cord prolapse and cord compression) would have occurred.

## Conclusion

This study shows the possibility of predicting birth asphyxia using a simple prediction model constructed from easily accessible and applicable maternal and neonatal characteristics, including premature rupture of the membrane, meconium aspiration, malpresentation, prolonged labor, preterm and tight nuchal cord). The derived score has good sensitivity for predicting birth asphyxia. It has n very good discriminative ability (accuracy). This new and relatively simple birth asphyxia risk score had a good prediction performance in Ethiopia. This is an important tool for predicting birth asphyxia.

Therefore, this score may prove to be a better model for application in low and middle-income countries. However, before introducing it to clinical and public health practices, further external validation (geographical validation) studies are needed to improve the prediction accuracy and applicability of the risk prediction tool. Our prediction model constitutes variables that are easily applicable and have reasonable accuracy to be used by both mid-and lower-level health professionals in each type of health institution. Findings from this study should be interpreted from the perspective of the following limitations. Frist limitation, as a single site (one zone) study, it is confined to a single area, which needs external validation before using it in another context. Second, pre-pregnancy body mass index, gestational weight gain, and maternal nutritional status were not assessed.

## Data Availability

All the data sets are available on the hand of the corresponding author.

## Abbreviations

APH: Antepartum hemorrhage
AUROC: Area Under the Receiver Operating Characteristics
LP: Linear prediction
MAS: Meconium aspiration syndrome
WHO: World Health Organization
